# Supplementary material for: BGCFlow: systematic pangenome workflow for the analysis of biosynthetic gene clusters across large genomic datasets
Source: Nucleic Acids Res. 2024 Apr 30;52(10):5478–95. doi: 10.1093/nar/gkae314 (PMC11162802; doi:10.1093/nar/gkae314)
Supplement: gkae314_Supplemental_File [file gkae314_supplemental_file.pdf]

Supplementary Information

BGCFlow: Systematic pangenome workflow for the analysis of biosynthetic gene clusters across large genomic datasets

Matin Nuhamunada[1]*, Omkar S. Mohite[1]*, Patrick V. Phaneuf[1], Bernhard O. Palsson[1,2#], Tilmann Weber[1#]

[1]The Novo Nordisk Foundation Center for Biosustainability, Technical University of Denmark, Kongens Lyngby 2800, Denmark.

[2]Department of Bioengineering, University of California San Diego, La Jolla, CA 92093, USA.

*These authors contributed equally.

[#]To whom correspondence should be addressed. Email: tiwe@biosustain.dtu.dk palsson@eng.ucsd.edu

Table of Contents

Table S1. List of 15 main computational tools to select the large-scale genome mining analysis using BGCFlow 1
Table S2. Rule names in BGCFlow main Snakemake workflow 2
Table S3. Additional sub-workflows available in BGCFlow 4
Figure S1. Project configuration and metadata to setup BGCFlow 5
Figure S2. Job scheduling and monitoring using Snakemake and panoptes 6
Figure S3. Entity Relations Diagram of the DuckDB OLAP database 7
Figure S4. Example of Jupyter-based markdown reports 8
Figure S5. Overview of timeline, quality, and taxonomic placement of 42 *Saccharopolyspora* genomes 9
Figure S6. Report of genome clustering using Mash distances. 10
Figure S7. Gene comparison of staphylobactin-like BGCs in *Saccharopolyspora* 11
Figure S8. Comparison of BiG-SCAPE network with different cutoffs assignment with the enriched network 13
Figure S9. Gene comparison of spinosyn-like BGCs in *Saccharopolyspora* 14
Figure S10. Median Beta-RD distribution of Saccharopolyspora GCFs 15
Figure S11. Co-Phylogenetic Analysis of representative BGC members of BiG-FAM model GCF_201888 17
Data S1. Input and result tables related to the PEP on qc_saccharaopolyspora 18
Data S2. Input and result tables related to the PEP on mq_saccharaopolyspora 18
Data S3. Results of BiG-FAM and ARTS database related to the PEP on mq_saccharaopolyspora 18
Data S4. Results of detailed GCF comparison related to the PEP on staphylobactin-like BGCs 18
Data S5. Results of detailed GCF comparison related to the PEP on spinosyn-like BGCs 18
Data S6. Results of detailed GCF comparison related to the PEP on erythreapeptin-like BGCs 18
Data S7. Results of detailed GCF comparison related to the PEP on mycofactocin-like BGCs 18
Data S8. Results of lsaBGC-easy pipeline 18
References 19

Table S1. List of 15 main computational tools to select the large-scale genome mining analysis using BGCFlow

	Rule Name	Tool Name	Version	Description	Link	Refs
1	eggnog	eggnog-mapper	2.1.6	Functional annotation of genome sequences using pre-computed Orthologous Group and phylogenies from the EggNOG database (http://eggnog5.embl.de).	https://github.com/eggnogdb/eggnog-mapper	(1, 2)
2	mash	mash	2.3	Calculate pairwise distance estimation for all samples using MinHash.	https://github.com/marbl/Mash	(3, 4)
3	fastani	fastani	1.33	Calculate pairwise Average Nucleotide Identity (ANI) across all samples.	https://github.com/ParBLiSS/FastANI	(5)
4	automlst_wrapper	Automlst-wapper	2bccf68	Simplified Species Tree building of all samples using [autoMLST](https://github.com/NBChub/automlst-simplified-wrapper)	https://github.com/KatSteinke/automlst-simplified-wrapper	(6)
5	roary	roary	3.13.0	Build pangenome from all samples using Roary.	https://github.com/sanger-pathogens/Roary	(7)
6	seqfu_stats	Seqfu2	1.15.3	Calculate sequence statistics using SeqFu.	https://github.com/telatin/seqfu2	(8)
7	bigslice	bigslice	ba4056a	Cluster BGCs using BiG-SLiCE (https://github.com/medema-group/bigslice) or map BGCs to BiG-FAM database (https://bigfam.bioinformatics.nl/)	https://github.com/medema-group/bigslice	(9, 10)
8	checkm	checkm-genome	1.1.3	Assess genome quality with CheckM.	https://github.com/Ecogenomics/CheckM	(11)
9	gtdbtk	gtdbtk	2.1.0	Taxonomic placement with GTDB-Tk with GTDB release 207	https://github.com/Ecogenomics/GTDBTk	(12, 13)
10	prokka	prokka	1.14.6	Copy annotated genbank results.	https://github.com/tseemann/prokka	(14)
11	antismash	antismash	6.1.1	Summarizes antiSMASH result.	https://github.com/antismash	(15)
12	arts	arts	3e32474	Targeted genome mining with Antibiotic Resistant Target Seeker (ARTS2) on samples.	https://github.com/NBChub/arts_v3	(16, 17)
13	deeptfactor	DeepTFactor	7f1bcb4	Use deep learning to find Transcription Factors.	https://bitbucket.org/kaistsystemsbiology/deeptfactor/src	(18)
14	bigscape	BiG-SCAPE	1.1.4	Cluster BGCs using BiG-SCAPE	https://github.com/medema-group/BiG-SCAPE	(19)
15	gecco	GECCO	0.9.10	Biosynthetic Gene Cluster prediction with Conditional Random Fields.	https://github.com/zellerlab/GECCO	(20)

Table S2. Rule names in BGCFlow main Snakemake workflow

node_number	Rule Name	Description	Refs
1	fix_gtdb_taxonomy	Gather and fix taxonomy metadata into a summary table	
2	gtdb_prep	Fetch taxonomic information from publicly available genomes using GTDB API	(21)
3	seqfu_combine	Combine all seqfu result into a table	
4	seqfu_stats	Calculate sequence statistics using SeqFu.	(8)
5	ncbi_genome_download	Download NCBI genome assemblies from RefSeq or GenBank using ncbi-genome-download (https://github.com/kblin/ncbi-genome-download)	
6	patric_genome_download	Fetch genome fasta files from Patric database	
7	copy_custom_fasta	Grab user-provided input fasta file for processing	
8	mash_convert	Convert MASH distance result into pandas ready matrix	
9	mash	Calculate pairwise distance estimation for all samples using MinHash.	(3, 4)
10	fastani_convert	Convert FastANI result into pandas ready matrix	
11	fastani	Calculate pairwise Average Nucleotide Identity (ANI) across all samples.	(5)
12	checkm_out	Extract and summarizes CheckM results	
13	checkm	Assess genome quality with CheckM.	(11)
14	install_checkm	Install CheckM locally	
15	evaluate_gtdbtk_input	Evaluate and validate input files for compatibility with GTDB-tk.	(12, 13)
16	prepare_gtdbtk_input	Prepare input files for GTDB-tk	
17	copy_prokka_gbk	Get a copy of annotated genbank files into processed folder	
18	format_gbk	Correct naming and add taxonomy metadata in annotated genbank files	
19	prokka	Annotate bacterial genomes with prodigal and Prokka	(14)
20	extract_meta_prokka	Extract any taxonomy information from fasta files	
21	antismash_summary	Correct shortened accession and compile antiSMASH summary into a table	
22	copy_log_changes	Copy and log changes in the workflow execution for audit and troubleshooting purposes.	
23	downstream_bgc_prep	Prepare region genbank files and metadata for downstream analysis	
24	antismash	Detection of Biosynthetic Gene Clusters with antiSMASH	(15, 22)
25	antismash_db_setup	Set up databases required for antiSMASH locally	
26	bgc_count	Summarizes BGC count of a given antiSMASH result	
27	copy_antismash	Generate symlinks of antiSMASH result in the processed folder	
28	antismash_overview_gather	Compile all antiSMASH summary pages into a table	
29	antismash_overview	Extract the antiSMASH summary page of a given antiSMASH result	
30	annotate_bigfam_hits	Annotate BiG-FAM database hits with additional metadata or functional information.	
31	summarize_bigslice_query	Summarize BiG-FAM GCF hits	

node_number	Rule Name	Description	Refs
32	query_bigslice	Map BGCs to BiG-FAM database (https://bigfam.bioinformatics.nl/)	(9, 10)
33	fetch_bigslice_db	Install BiG-FAM database locally	
34	copy_bigscape	Copy and format BiG-SCAPE result into processed folder	
35	bigscape	Cluster BGCs using BiG-SCAPE	(19)
36	install_bigscape	Install BiG-SCAPE locally	
37	bigscape_to_cytoscape	Generate cytoscape ready tables and annotation files	
38	get_mibig_table	Get MIBIG 3.0 tables locally	(23)
39	bigslice	Cluster BGCs using BiG-SLiCE (https://github.com/medema-group/bigslice)	(10)
40	bigslice_prep	Prepare files and metadata required for BiG-SLICE clustering	
41	automlst_wrapper_out	Extract and format autoMLST tree into processed folder	
42	automlst_wrapper	Simplified Species Tree building of all samples using [autoMLST](https://github.com/NBChub/automlst-simplified-wrapper)	(6)
43	prep_automlst_gbk	Prepare input files for autoMLST	
44	install_automlst_wrapper	Install autoMLST locally	
45	arts_final	Compile final ARTS analysis results and generate summary reports.	
46	arts_allhits_combine	Combine all ARTS hits across samples into a comprehensive summary table.	
47	arts_extract	Extract BGC proximity hits summary from an ARTS result	
48	arts	Targeted genome mining with Antibiotic Resistant Target Seeker (ARTS2) on samples.	(16, 17)
49	arts_bgctable_combine	Aggregate BGC-related results from ARTS analysis into a consolidated table.	
50	arts_coretable_combine	Combine core model hits from ARTS into a single table for easy access and interpretation.	
51	arts_knownhits_combine	Compile known resistance hits from ARTS analysis into a unified table for further examination.	
52	roary_out	Extract ROARY information	
53	roary	Build pangenome from all samples using Roary (https://github.com/sanger-pathogens/Roary).	(7)
54	eggnog	Functional annotation of genome sequences using pre-computed Orthologous Group and phylogenies from the EggNOG database (http://eggnog5.embl.de).	(1, 2)
55	install_eggnog	Install Eggnog databases locally	
56	eggnog_roary_result_copy	Copy results from eggNOG mapper analysis of Roary outputs for downstream processing.	
57	eggnog_roary	Functional annotation of Roary output using eggNOG mapper	(1, 2, 7)
58	roary_reassign_pangene_categories	Reassign categories to pangenome elements based into predefined groups.	
59	deeptfactor_summary	Combine all DeepTFactor result into a table	
60	deeptfactor_to_json	Convert DeepTFactor result into json table	
61	deeptfactor	Use deep learning to find Transcription Factors.	(18)
62	deeptfactor_setup	Install DeepTFactor locally	
63	deeptfactor_roary	Use DeepTFactor on Roary outputs.	(7, 18)

node_number	Rule Name	Description	Refs
64	cblaster_genome_db	Build diamond database of genomes for cblaster search.	(24, 25)
65	cblaster_bgc_db	Build diamond database of BGCs for cblaster search.	(24, 25)
66	gecco_aggregate	Aggregate results from GECCO analysis into a comprehensive summary for further analysis.	
67	gecco	Predict BGCs using GECCO (https://github.com/zellerlab/GECCO).	(20)
68	extract_ncbi_information	Capture metadata of downloaded genomes from NCBI	
69	csv_to_parquet	Convert CSV files into Parquet format for efficient storage and faster access.	
70	copy_mibig_table	Copy MIBIG database tables into the workflow for reference or downstream analysis.	

Table S3. Additional sub-workflows available in BGCFlow

	BGCFlow Sub-workflow (snakefiles)	Description	References
1	BGC (workflow/BGC)	Comparative BGC analysis of a given antiSMASH region genbanks	(26–28)
2	Database (workflow/Database)	Build a DuckDB OLAP database from BGCFlow run results	(29)
3	Report (workflow/Report)	Build an interactive markdown reports from Jupyter notebook templates	
4	Metabase (workflow/Metabase)	Install and serve Metabase with DuckDB plugins for interactive exporation and visualization of OLAP database generated by BGCFlow	
5	lsaBGC (workflow/lsabgc)	Run lsabgc-easy pipeline for evolutionary and population genetics of BGCs	(30)
6	Ppanggolin (workflow/ppanggolin)	Build a pangenome graph and detect region of genomic plasticity	(31)
7	Alleleome (workflow/Allelome)	Explore and analyze natural sequence variations within the Open Reading Frames (ORFs) of alleles of core genes in a species pan-genome	(32)

a)

```yaml
# This file should contain everything to configure the workflow on a global scale.

#### PROJECT INFORMATION ####
# This section control your project configuration.
# Each project are separated by "-".
# A project must contain the variable "name" and "samples".
# - name : name of your project
# - samples : a csv file containing a list of genome ids for analysis with multiple sources mentioned. Genome ids must be unique.
# - prokka-db (optional): list of the custom accessions to use as prokka reference database
# - gtdb-tax (optional): output summary file of GTDB-tk with "user_genome" and "classification" as the two minimum columns

projects:
# Project 1
- name: config/qc_saccharopolyspora/project_config.yaml

# Project 2
 - name: config/mq_saccharopolyspora/project_config.yaml

#### RESOURCES CONFIGURATION ####
# resources : the location of the resources to run the rule.
# The default location is at "resources/{resource_name}".
resources_path:
  antismash_db: resources/antismash_db
  eggnog_db: resources/eggnog_db
  BiG-SCAPE: resources/BiG-SCAPE
  bigslice: resources/bigslice
  checkm: resources/checkm
  #RNAmmer: resources/RNAmmer # Path must be specified if rule configuration "rnammer" is TRUE
```

b)

```yaml
name: mq_saccharopolyspora
pep_version: 2.1.0
description: '28 Saccharopolysopra genomes of medium to high quality with less than 60 contigs'
sample_table: samples.csv
prokka-db: prokka-db.csv
gtdb-tax: 'gtdbtk.bac120.summary.tsv'

rules:
  seqfu: TRUE
  mash: TRUE
  fastani: TRUE
  checkm: FALSE
  gtdbtk: FALSE
  prokka-gbk: FALSE
  antismash: TRUE
  query-bigslice: TRUE
  bigscape: TRUE
  bigslice: FALSE
  automlst-wrapper: TRUE
  arts: TRUE
  roary: FALSE
  eggnog: FALSE
  eggnog-roary: FALSE
  deeptfactor: FALSE
  deeptfactor-roary: FALSE
  cblaster-genome: FALSE
  cblaster-bgc: FALSE
```

c)

1	genome_id	source	organism	genus	species	strain
2	GCF_003635025.1	ncbi				
3	GCF_900114905.1	ncbi				
4	GCF_007829955.1	ncbi				
5	GCF_018070075.1	ncbi				
6	GCF_016859185.1	ncbi				
7	GCF_018141105.1	ncbi				
8	GCF_022392385.1	ncbi				
9	GCF_000062885.1	ncbi				
10	GCF_002564065.1	ncbi				
11	GCF_900116135.1	ncbi				
12	GCF_014203325.1	ncbi				
13	GCF_022828475.1	ncbi				
14	GCF_024734405.1	ncbi				
15	GCF_008630535.1	ncbi				
16	GCF_013410345.1	ncbi				
17	GCF_900112555.1	ncbi				
18	GCF_900108315.1	ncbi				
19	GCF_014203395.1	ncbi				
20	GCF_000710755.1	ncbi				
21	GCF_003931915.1	ncbi				
22	GCF_025643595.1	ncbi				
23	GCF_012277335.1	ncbi				
24	GCF_015209505.1	ncbi				
25	GCF_016526145.1	ncbi				
26	GCF_014490055.1	ncbi				
27	GCF_002846475.1	ncbi				
28	GCF_000194155.1	ncbi				
29	GCF_014646075.1	ncbi				

d)

1	Accession	Strain Description
2	GCA_000062885.1	Saccharopolyspora erythraea NRRL 2338 (high GC Gram+)
3	GCA_000009765.2	Streptomyces avermitilis MA-4680 = NBRC 14893 (high GC Gram+)
4	GCA_000010605.1	Streptomyces griseus subsp. griseus NBRC 13350 (high GC Gram+)
5	GCA_000015005.1	Mycolicibacterium smegmatis MC2 155 (high GC Gram+)
6	GCA_000196835.1	Amycolatopsis mediterranei U32 (high GC Gram+)
7	GCA_000203835.1	Streptomyces coelicolor A3(2) (high GC Gram+)
8	GCA_000444875.1	Streptomyces collinus Tu 365 (high GC Gram+)
9	GCA_000739105.1	Streptomyces lividans TK24 (high GC Gram+)

Figure S1. Project configuration and metadata to setup BGCFlow

a) Global configuration file (*yaml*) with path to two of the PEPs used in the demonstration. b) Example of the *mq_saccharopolyspora* PEP configuration file with selected rules to run. c) List of 28 NCBI accession IDs in the samples table used in the project (*samples.csv*). d) List of selected high-quality genome annotations of actinomycetes as priority of prokka annotation. (*prokka-db.csv*)

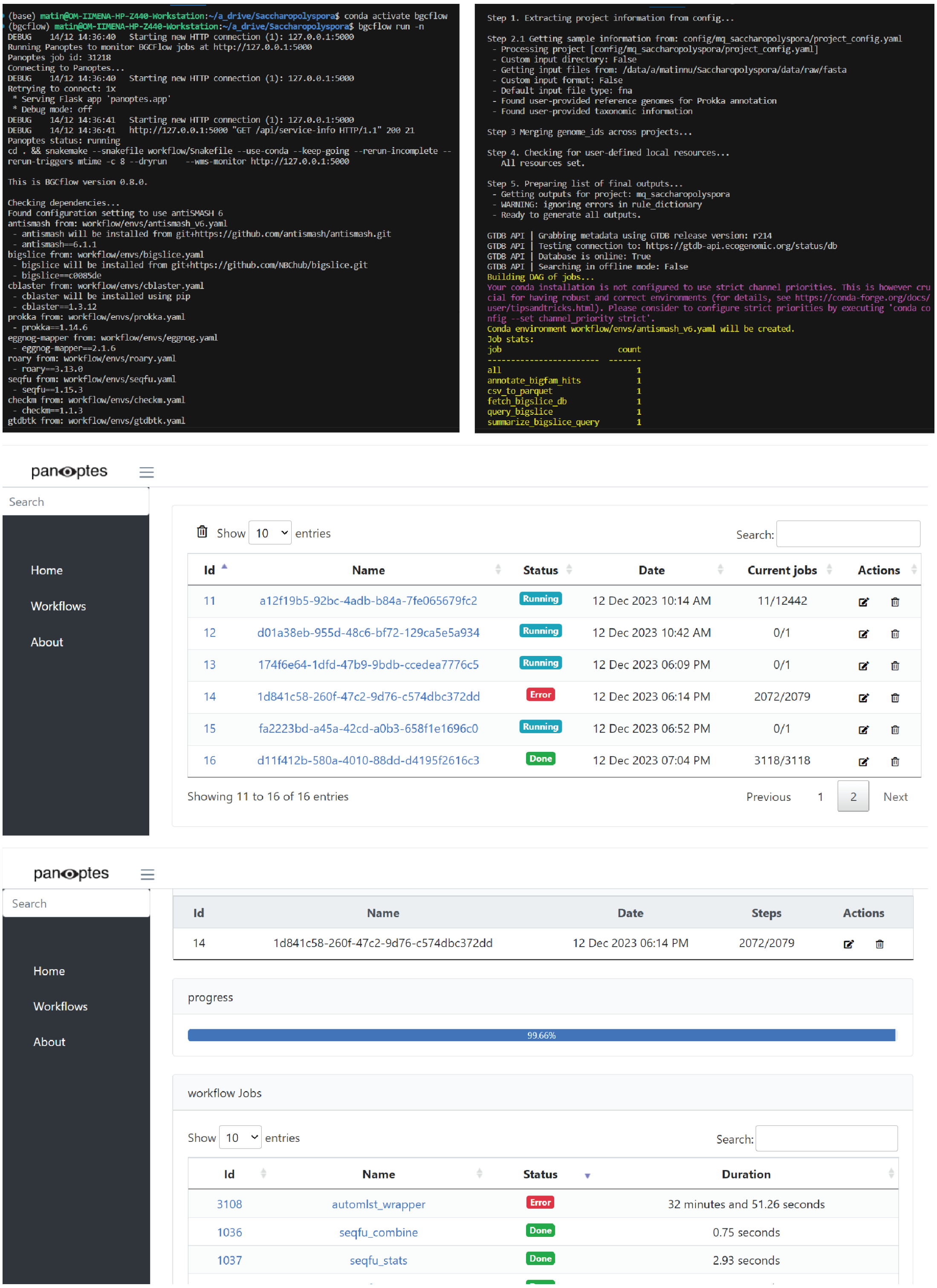

Figure S2. Job scheduling and monitoring using Snakemake and panoptes
Screenshot of the dry run for BGCFlow on the *mq_saccharopolyspora* with all jobs to run for the selected rules. The number of minimum threads for each jobs can be updated using configure file for the thread profiling. Also shown the monitoring of the Snakemake runs and individual jobs using panoptes.

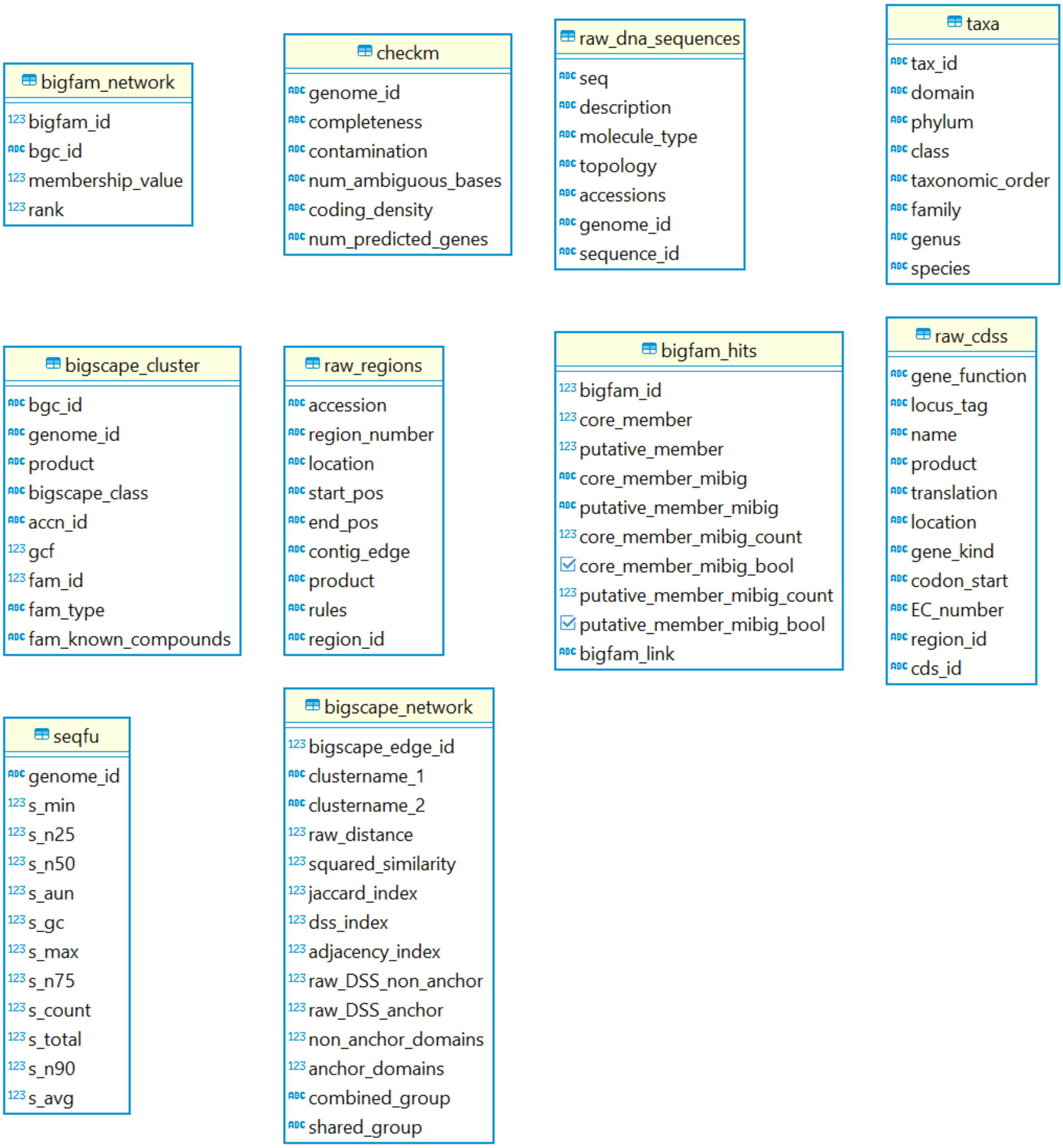

Figure S3. Entity Relations Diagram of the DuckDB OLAP database

Entity relations diagram of the exported DuckDB tables using DBT. Schema are being maintained in https://github.com/NBChub/bgcflow_dbt-duckdb.

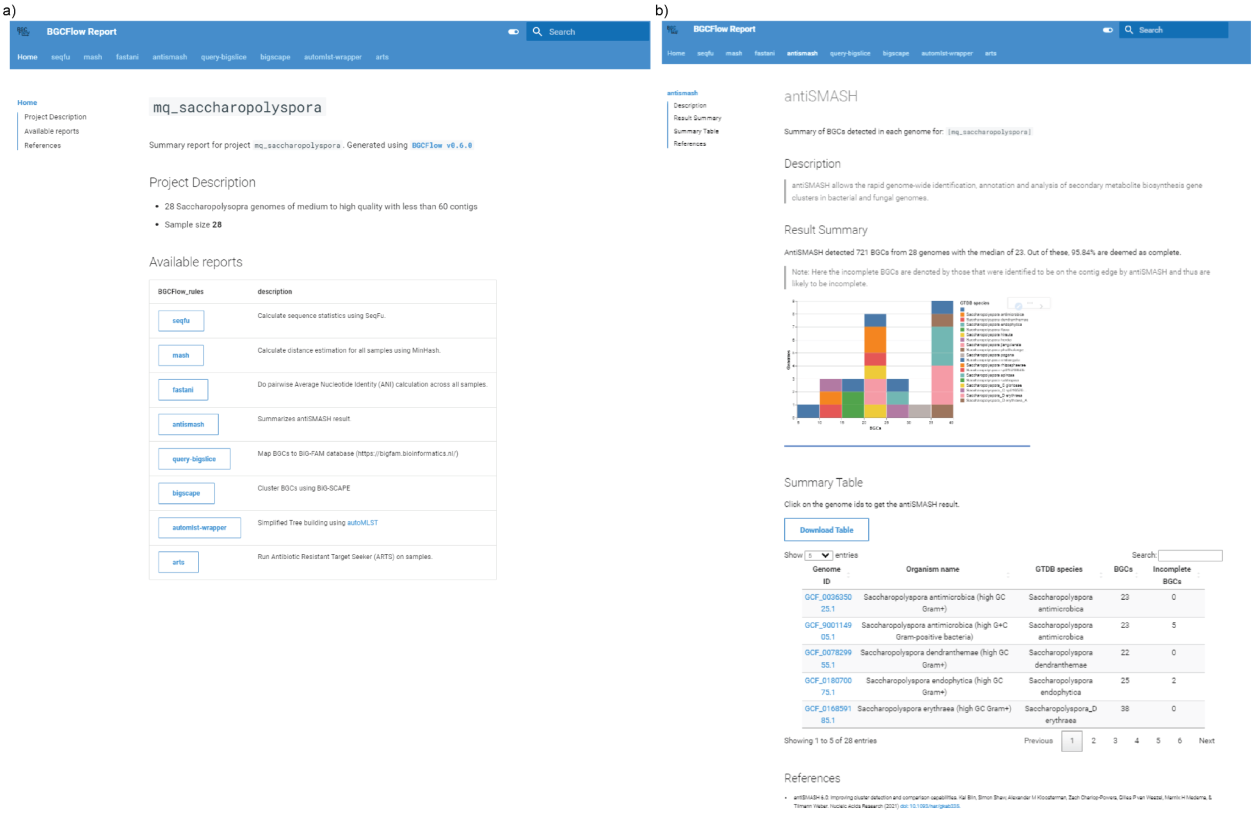

Figure S4. Example of Jupyter-based markdown reports

a) Screenshot of the home page of the BGCFlow reports of the *mq_saccharopolyspora* project with selected rules. b) Example report of the *antismash* rule's report page with a summary and the table with a list of genomes. The links for the genomes IDs in the table correspond to antiSMASH-generated HTML reports with details on all detected BGCs.

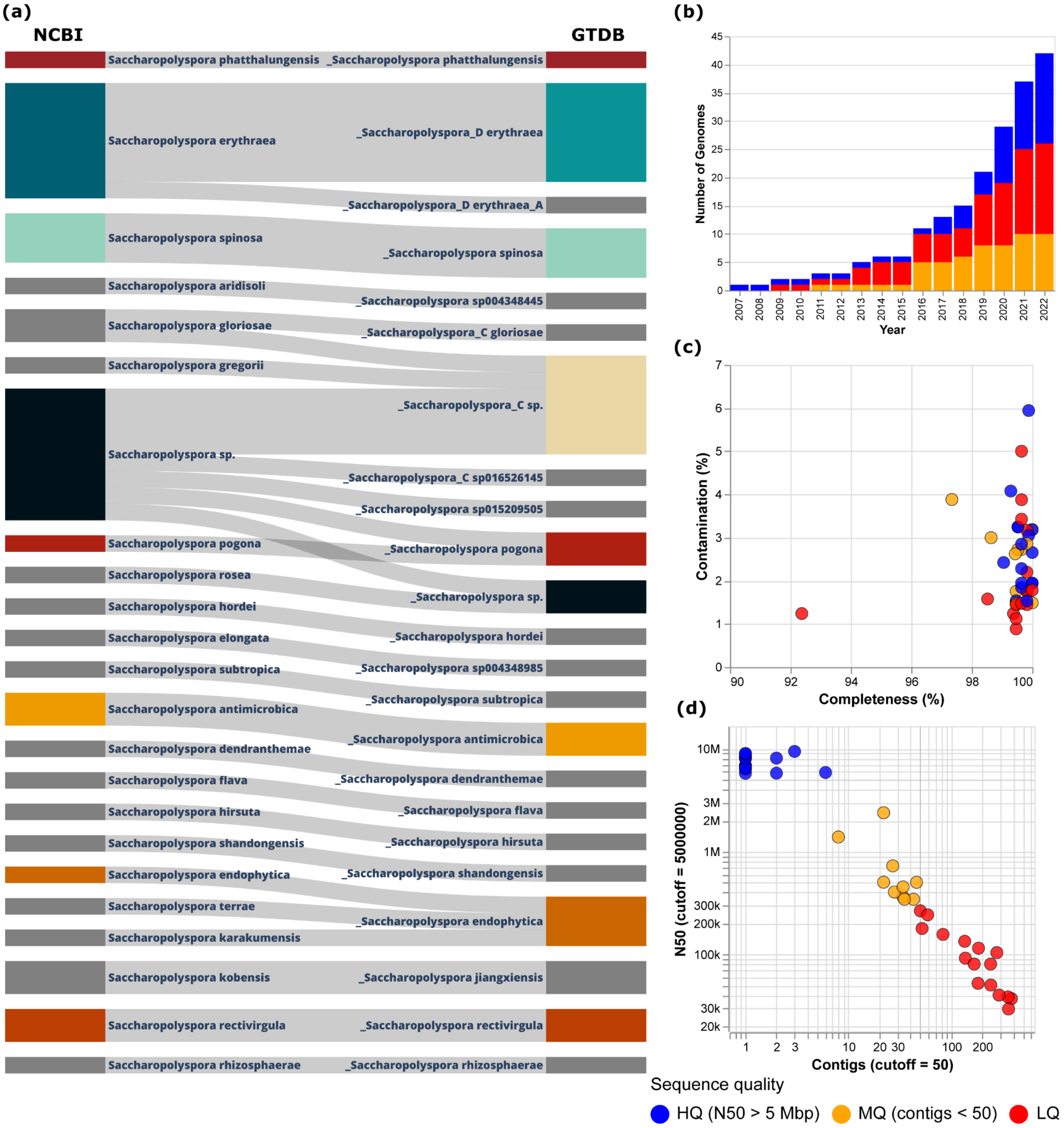

Figure S5. Overview of timeline, quality, and taxonomic placement of 42 *Saccharopolyspora* genomes

a) Cumulative bar chart of the number of genomes over the last 15 years with different assembly qualities. b) Distribution of contamination vs completeness metrics calculated using CheckM, where colors represent the assembly qualities. c) Sankey diagram representing the species assignment differences between NCBI and GTDB. d) Scatterplot representing the distribution of N50 values vs the number of contigs. The cutoff of 50 contigs is used to filter the low-quality genomes, whereas 5 Mbp of N50 value cutoff was used to define high-quality genomes. The remaining genomes were defined as medium-quality.

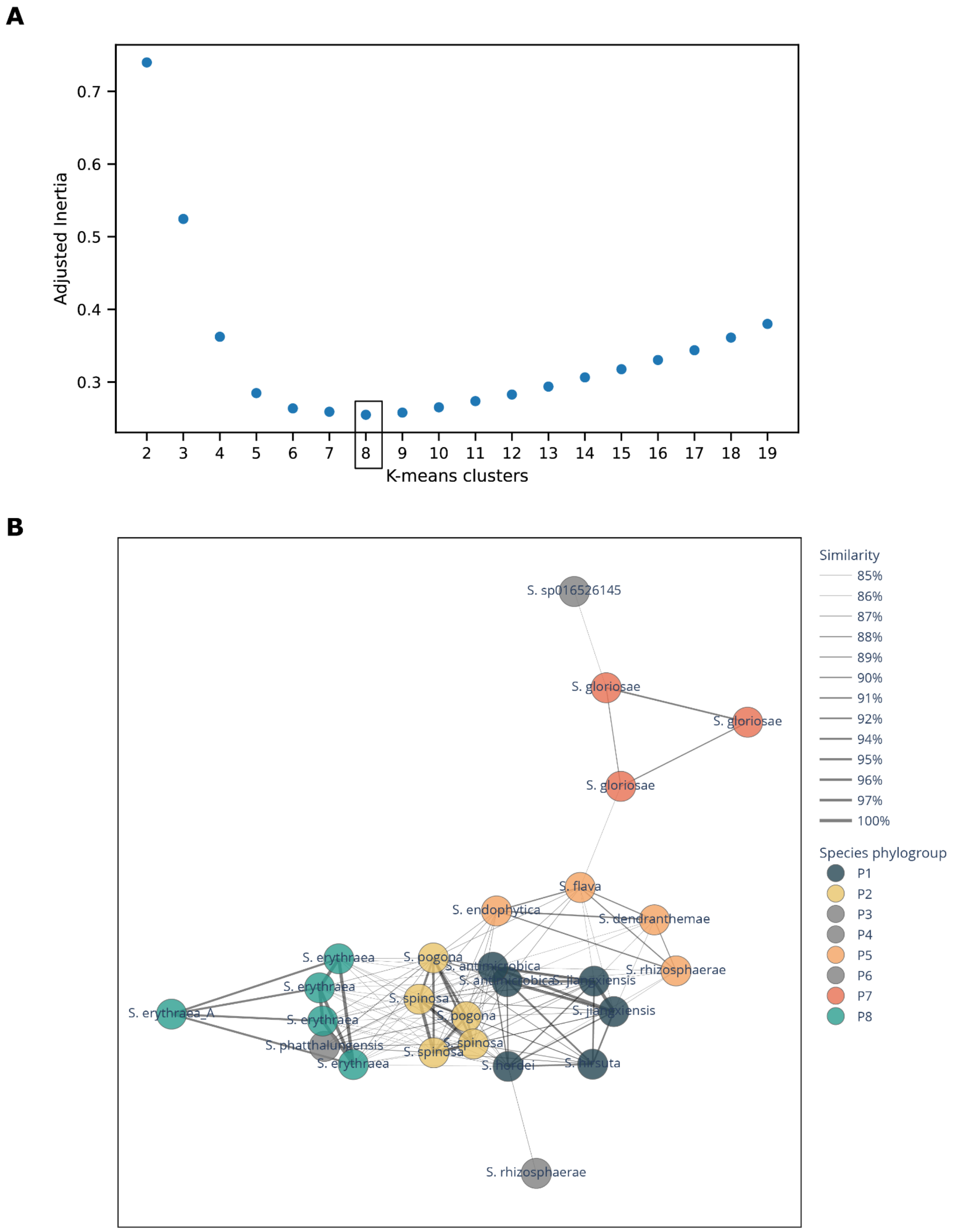

Figure S6. Report of genome clustering using Mash distances.

(a) Suggested optimal clustering based on K-means clustering with minimum adjusted inertia. (b) MASH-similarity network with nodes represented by genomes from different species and edge width represented by the similarity of 85% to 100%. The 8 optimal clusters are represented by different colors of nodes.

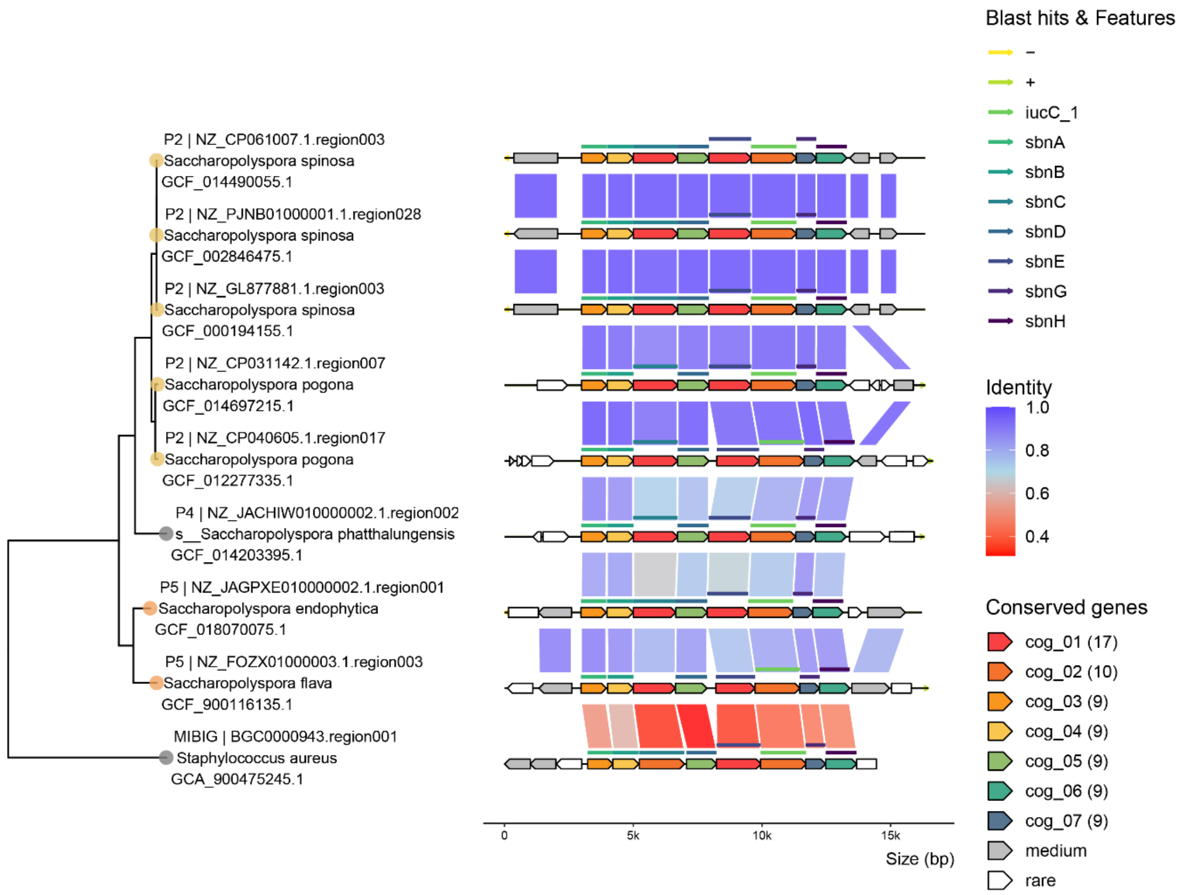

Figure S7. Gene comparison of staphylobactin-like BGCs in *Saccharopolyspora*
Comparison of staphylobactin-like BGCs which are connected through a BiG-FAM node *GCF_201888,* which has a Shannon index of ~0.3 and contained 12,444 BGCs which are distributed across 43 genera with the majority belonging to Staphylococcus (~94.2%). All 8 genes responsible for staphylobactin biosynthesis can be mapped through CBlaster except for NZ_FOZX01000003, with only 7 matches.

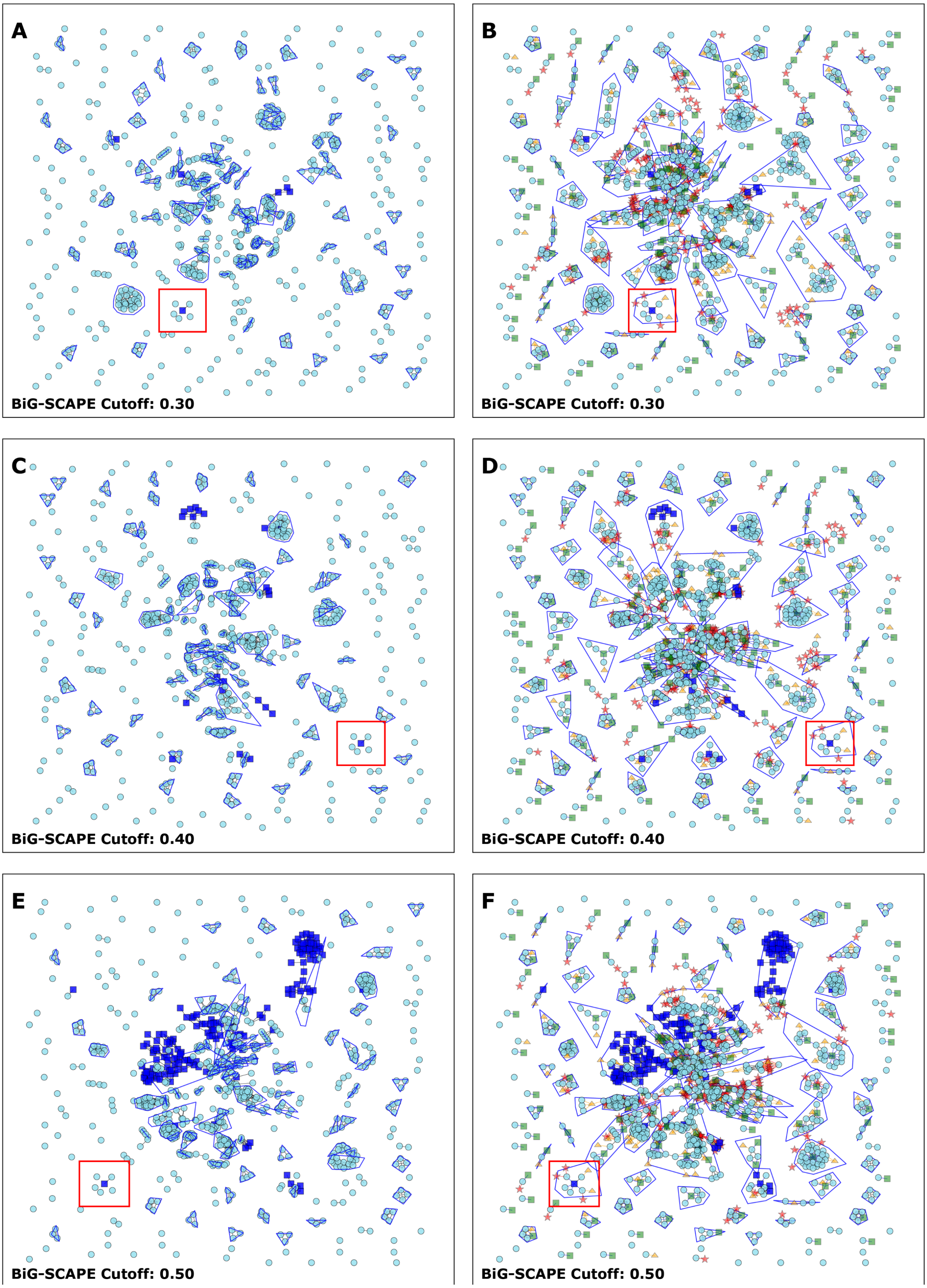
A
BiG-SCAPE Cutoff: 0.30
B
BiG-SCAPE Cutoff: 0.30
C
BiG-SCAPE Cutoff: 0.40
D
BiG-SCAPE Cutoff: 0.40
E
BiG-SCAPE Cutoff: 0.50
F
BiG-SCAPE Cutoff: 0.50

Figure S8. Comparison of BiG-SCAPE network with different cutoffs assignment with the enriched network

This figure compares the number of connected component assignments between an enriched and non-enriched BiG-SCAPE network with different cutoffs. BiG-SCAPE networks were enriched with ARTS2 hits, BiG-FAM hits, and KnownClusterBlast hits. BiG-FAM models 202087, 200946, 210179, 213140, 201682, 201608, 205957, 215277, 201830, and 202082 were removed because the assigned top genus in the model is below 30%. A) BiG-SCAPE sequence similarity network with cutoff 0.3 resulting in 328 connected components (GCFs) with 206 singletons. A total of 4 GCFs with 14 BGCs can be assigned to 6 MIBIG entries. B) Enriched BiG-SCAPE network with cutoff 0.3 resulting in 202 connected components with 44 singletons. This increases the number of BGCs in the 4 BiG-SCAPE GCF with MIBIG hits into 29 BGCs. Additionally, a total of 105 BGCs can be assigned to 12 GCFs with MIBIG KnownClusterBlast similarity >= 80% and left only 122 BGCs in 70 GCFs without connection to MIBIG and BiG-FAM nodes. C) BiG-SCAPE sequence similarity network with cutoff 0.4 resulting in 280 connected components (GCFs) with 162 singletons. A total of 10 GCFs with 80 BGCs can be assigned to 19 MIBIG entries. D) Enriched BiG-SCAPE network with cutoff 0.4 resulting in 190 connected components with 41 singletons. This increases the number of BGCs in the 10 BiG-SCAPE GCF with MIBIG hits into 109 BGCs. Additionally, a total of 53 BGCs can be assigned to 8 GCFs with MIBIG KnownClusterBlast similarity >= 80% and left only 115 BGCs in 64 GCFs without connection to MIBIG and BiG-FAM nodes. E) BiG-SCAPE sequence similarity network with cutoff 0.5 resulting in 259 connected components (GCFs) with 144 singletons. A total of 15 GCFs with 115 BGCs can be assigned to 110 MIBIG entries. F) Enriched BiG-SCAPE network with cutoff 0.5 resulting in 173 connected components with 36 singletons. This increases the number of BGCs in the 15 BiG-SCAPE GCF with MIBIG hits into 184 BGCs. Additionally, a total of 44 BGCs can be assigned to 7 GCFs with MIBIG KnownClusterBlast similarity >= 80% and left only 114 BGCs in 59 GCFs without connection to MIBIG and BiG-FAM nodes. Red box highlights the location of spinosyn-like BGCs in the network.

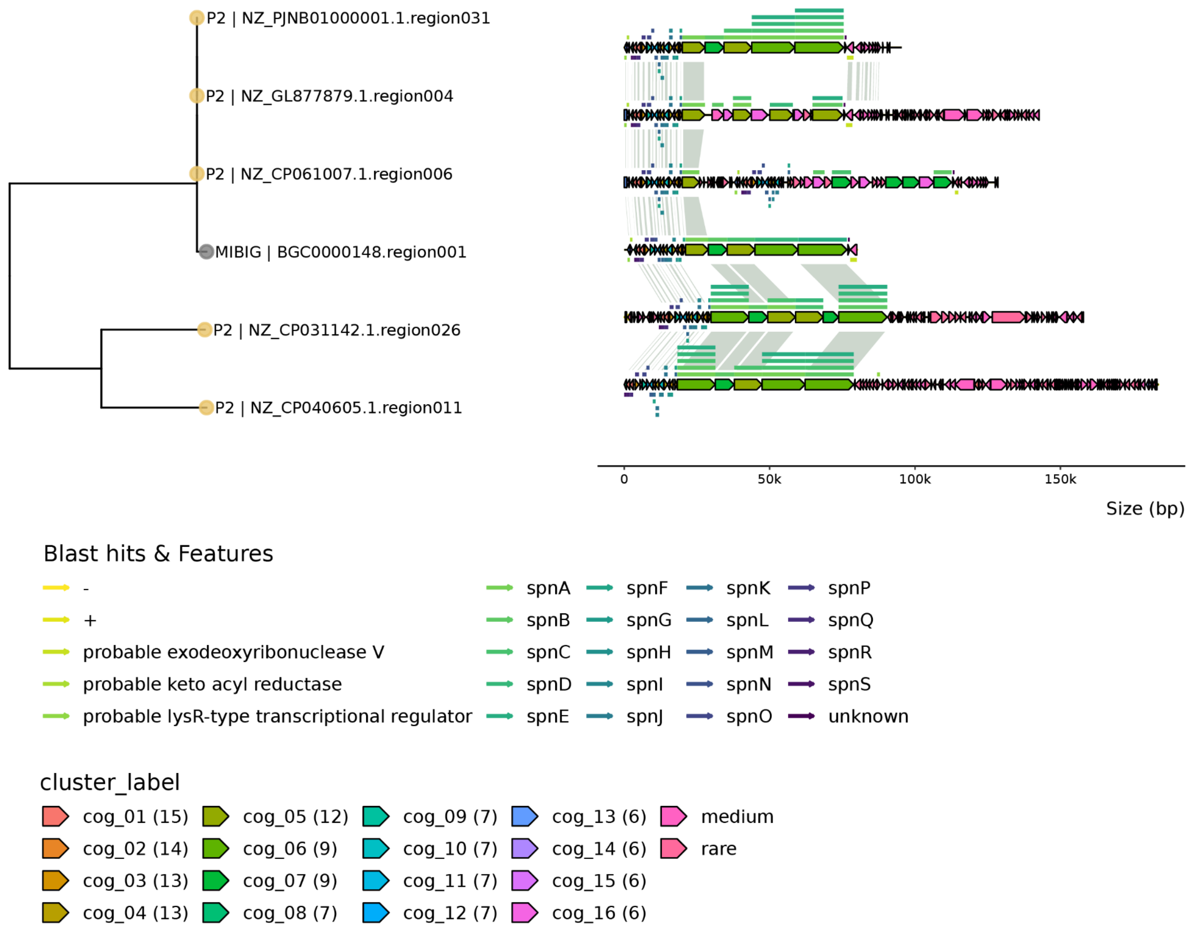

Figure S9. Gene comparison of spinosyn-like BGCs in *Saccharopolyspora*

Comparison of BGCs with BiG-SCAPE and KnownClusterBlast similarity to spinosyn. All regions matched with spinosyn belong to phylogroup 2. Of the 5 BGCs, only 1 has an exact structure with spinosyn MIBIG BGC (NZ_PJNB01000001). Other BGCs showed variation in the PKS modules and might even lose the whole biosynthesis pathway (NZ_CP061007). Annotations in all BGCs showed matches to specific tailoring enzymes found in spinosyn biosynthesis for sugar attachment (cog_03 - probable rhamonsyltransferase).

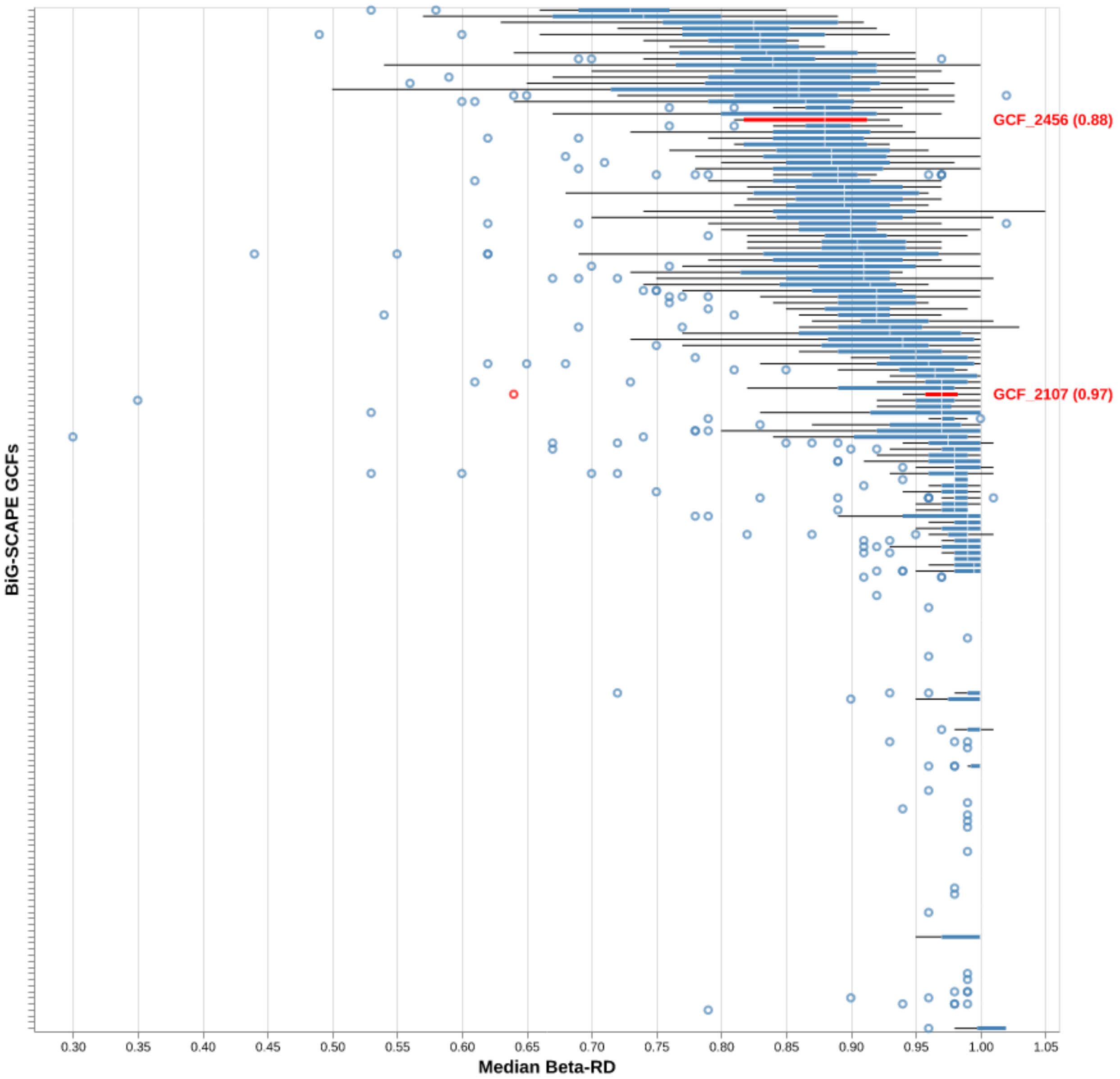

Figure S10. Median Beta-RD distribution of Saccharopolyspora GCFs

Summary of median Beta-RD distribution of BiG-SCAPE GCFs in Saccharopolyspora calculated using lsabgc (30). Only GCFs with non-null values are shown. Highlighted in red are GCFs with similarity to Staphylobactin (Staphyloferrin B) based on query result to BiG-FAM GCF model GCF_201888. Detailed beta-rd distribution and population genetic analysis is available from Data S8.

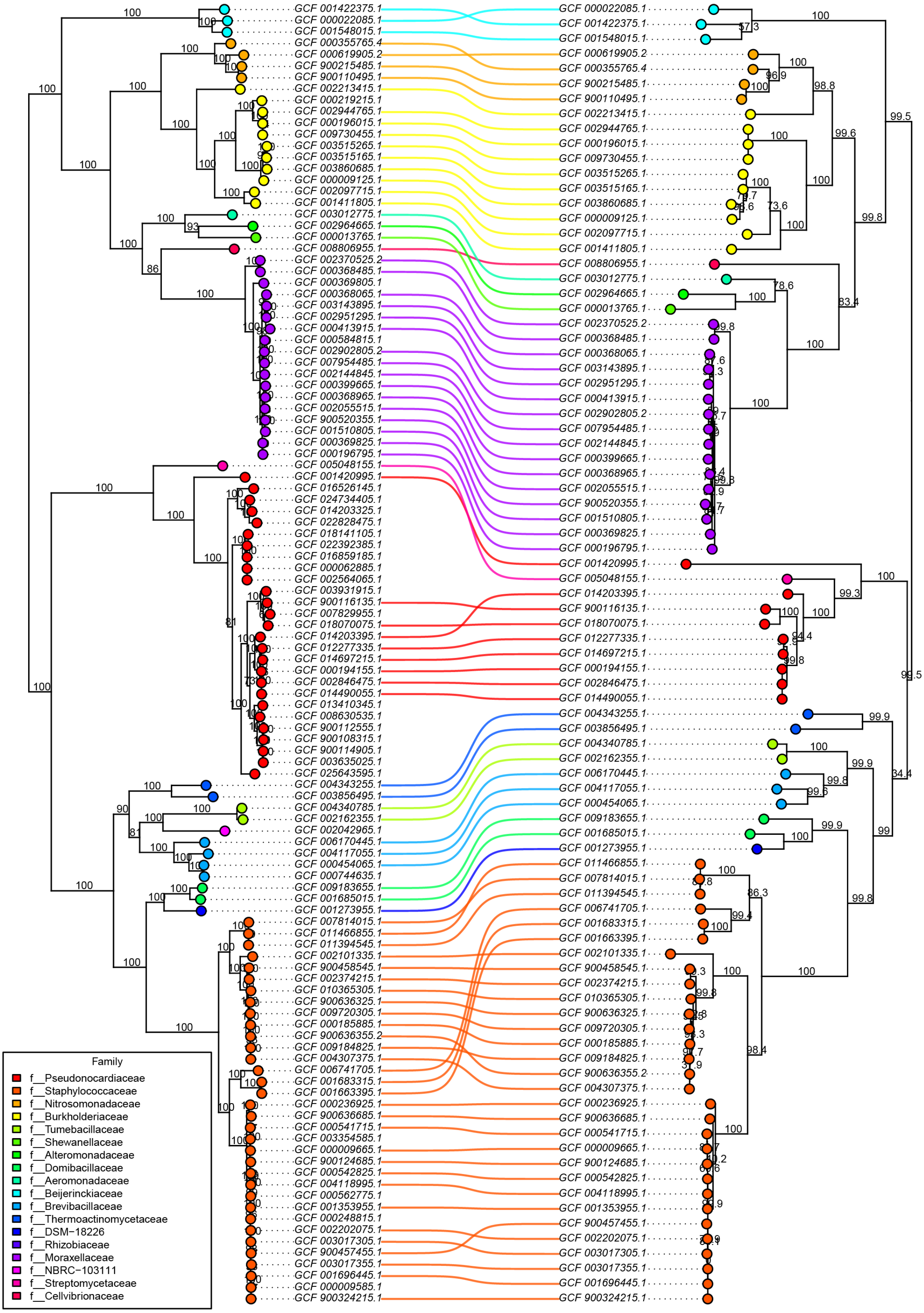
GCF 001422375.1
GCF 000022085.1
GCF 001548015.1
GCF 000355765.4
GCF 000619905.2
GCF 900215485.1
GCF 900110495.1
GCF 002213415.1
GCF 000219215.1
GCF 002944765.1
GCF 000196015.1
GCF 009730455.1
GCF 003515265.1
GCF 003515165.1
GCF 003860685.1
GCF 000009125.1
GCF 002097715.1
GCF 001411805.1
GCF 003012775.1
GCF 002964665.1
GCF 000013765.1
GCF 008806955.1
GCF 002370525.2
GCF 000368485.1
GCF 000369805.1
GCF 000368065.1
GCF 003143895.1
GCF 002951295.1
GCF 000413915.1
GCF 000584815.1
GCF 002902805.2
GCF 007954485.1
GCF 002144845.1
GCF 000399665.1
GCF 000368965.1
GCF 002055515.1
GCF 900520355.1
GCF 001510805.1
GCF 000369825.1
GCF 000196795.1
GCF 005048155.1
GCF 001420995.1
GCF 016526145.1
GCF 024734405.1
GCF 014203325.1
GCF 022828475.1
GCF 018141105.1
GCF 022392385.1
GCF 016859185.1
GCF 000062885.1
GCF 002564065.1
GCF 003931915.1
GCF 900116135.1
GCF 007829955.1
GCF 018070075.1
GCF 014203395.1
GCF 012277335.1
GCF 014697215.1
GCF 000194155.1
GCF 002846475.1
GCF 014490055.1
GCF 013410345.1
GCF 008630535.1
GCF 900112555.1
GCF 900108315.1
GCF 900114905.1
GCF 003635025.1
GCF 025643595.1
GCF 004343255.1
GCF 003856495.1
GCF 004340785.1
GCF 002162355.1
GCF 002042965.1
GCF 006170445.1
GCF 004117055.1
GCF 000454065.1
GCF 000744635.1
GCF 009183655.1
GCF 001685015.1
GCF 001273955.1
GCF 007814015.1
GCF 011466855.1
GCF 011394545.1
GCF 002101335.1
GCF 900458545.1
GCF 002374215.1
GCF 010365305.1
GCF 900636325.1
GCF 009720305.1
GCF 000185885.1
GCF 900636355.2
GCF 009184825.1
GCF 004307375.1
GCF 006741705.1
GCF 001683315.1
GCF 001663395.1
GCF 000236925.1
GCF 900636685.1
GCF 000541715.1
GCF 003354585.1
GCF 000009665.1
GCF 900124685.1
GCF 000542825.1
GCF 004118995.1
GCF 000562775.1
GCF 001353955.1
GCF 000248815.1
GCF 002202075.1
GCF 003017305.1
GCF 900457455.1
GCF 003017355.1
GCF 001696445.1
GCF 000009585.1
GCF 900324215.1
GCF 000022085.1
GCF 001422375.1
GCF 001548015.1
GCF 000619905.2
GCF 000355765.4
GCF 900215485.1
GCF 900110495.1
GCF 002213415.1
GCF 002944765.1
GCF 000196015.1
GCF 009730455.1
GCF 003515265.1
GCF 003515165.1
GCF 003860685.1
GCF 000009125.1
GCF 002097715.1
GCF 001411805.1
GCF 008806955.1
GCF 003012775.1
GCF 002964665.1
GCF 000013765.1
GCF 002370525.2
GCF 000368485.1
GCF 000368065.1
GCF 003143895.1
GCF 002951295.1
GCF 000413915.1
GCF 002902805.2
GCF 007954485.1
GCF 002144845.1
GCF 000399665.1
GCF 000368965.1
GCF 002055515.1
GCF 900520355.1
GCF 001510805.1
GCF 000369825.1
GCF 000196795.1
GCF 001420995.1
GCF 005048155.1
GCF 014203395.1
GCF 900116135.1
GCF 018070075.1
GCF 012277335.1
GCF 014697215.1
GCF 000194155.1
GCF 002846475.1
GCF 014490055.1
GCF 004343255.1
GCF 003856495.1
GCF 004340785.1
GCF 002162355.1
GCF 006170445.1
GCF 004117055.1
GCF 000454065.1
GCF 009183655.1
GCF 001685015.1
GCF 001273955.1
GCF 011466855.1
GCF 007814015.1
GCF 011394545.1
GCF 006741705.1
GCF 001683315.1
GCF 001663395.1
GCF 002101335.1
GCF 900458545.1
GCF 002374215.1
GCF 010365305.1
GCF 900636325.1
GCF 009720305.1
GCF 000185885.1
GCF 009184825.1
GCF 900636355.2
GCF 004307375.1
GCF 000236925.1
GCF 900636685.1
GCF 000541715.1
GCF 000009665.1
GCF 900124685.1
GCF 000542825.1
GCF 004118995.1
GCF 001353955.1
GCF 900457455.1
GCF 002202075.1
GCF 003017305.1
GCF 003017355.1
GCF 001696445.1
GCF 900324215.1
Family
f__Pseudonocardiaceae
f__Staphylococcaceae
f__Nitrosomonadaceae
f__Burkholderiaceae
f__Tumebacillaceae
f__Shewanellaceae
f__Alteromonadaceae
f__Domibacillaceae
f__Aeromonadaceae
f__Beijerinckiaceae
f__Brevibacillaceae
f__Thermoactinomycetaceae
f__DSM−18226
f__Rhizobiaceae
f__Moraxellaceae
f__NBRC−103111
f__Streptomycetaceae
f__Cellvibrionaceae

Figure S11. Co-Phylogenetic Analysis of representative BGC members of BiG-FAM model GCF_201888

Phylogenetic comparison of genome tree (right) built using AutoMLST (6) and BGC tree (left) built using getphylo (26). The tanglegram was built using the R package Paco (33), with colors highlighting the family of where the genome/BGCs belongs to. Genomes of Saccharopolyspora which do not have the Staphylobactin-like BGCs are also included. The three orthologs chosen by getphylo encodes for the gene annotated for Staphyloferrin B transporter, N-((2S)-2-amino-2-carboxyethyl)-L-glutamate dehydrogenase, and N-(2-amino-2-carboxyethyl)-L-glutamate synthase.

Data S1. Input and result tables related to the PEP on qc_saccharaopolyspora
Tab 1. Content of the sample.csv listing 42 NCBI genomes as input for the BGCFlow PEP configuration
Tab 2. NCBI metadata of the 42 NCBI genomes
Tab 3. CheckM results on quality assessment of the 42 NCBI genomes
Tab 4. SeqFu results on quality assessment of the 42 NCBI genomes
Tab 5. GTDB-tk and GTDB results on taxonomic definition of the 42 NCBI genomes

Data S2. Input and result tables related to the PEP on mq_saccharaopolyspora
Tab 1. Content of the sample.csv listing 26 genomes as input for the BGCFlow PEP configuration
Tab 2. MASH distance of the selected 26 genomes
Tab 3. MASH-base phlyogroups of the selected 26 genomes
Tab 4. Prokka annotation result summary of the selected 26 genomes
Tab 5. List of BGC hits from antiSMASH
Tab 6. Results of GCFs based on BiG-SCAPE using 0.3 cutoff
Tab 7. Results of GCFs based on BiG-SCAPE using 0.4 cutoff
Tab 8. Results of GCFs based on BiG-SCAPE using 0.5 cutoff
Tab 9. Edge table of BiG-SCAPE sequence similarity network using 0.3 cutoff
Tab 10. Edge table of BiG-SCAPE sequence similarity network using 0.4 cutoff
Tab 11. Edge table of BiG-SCAPE sequence similarity network using 0.5 cutoff

Data S3. Results of BiG-FAM and ARTS database related to the PEP on mq_saccharaopolyspora
Tab 1. Hits against the BiG-FAM GCFs calculated using BiG-SLICE query
Tab 2. List of BiG-FAM GCFs hits
Tab 3. Hits against ARTS profile
Tab 4. Table with all edges represented in the enriched network with BiG-SCAPE cutoff 0.3

Data S4. Results of detailed GCF comparison related to the PEP on staphylobactin-like BGCs
Tab 1. CDS Feature and COG annotations of staphylobactin-like GCF
Tab 2. Clinker links for BGC alignment of staphylobactin-like GCF
Tab 3. CBlaster hits for BGC alignment of staphylobactin-like GCF

Data S5. Results of detailed GCF comparison related to the PEP on spinosyn-like BGCs
Tab 1. CDS Feature and COG annotations of spinosyn-like GCF
Tab 2. Clinker links for BGC alignment of spinosyn-like GCF
Tab 3. CBlaster hits for BGC alignment of spinosyn-like GCF

Data S6. Results of detailed GCF comparison related to the PEP on erythreapeptin-like BGCs
Tab 1. CDS Feature and COG annotations of erythreapeptin-like GCF
Tab 2. Clinker links for BGC alignment of erythreapeptin-like GCF
Tab 3. CBlaster hits for BGC alignment of erythreapeptin-like GCF

Data S7. Results of detailed GCF comparison related to the PEP on mycofactocin-like BGCs
Tab 1. CDS Feature and COG annotations of mycofactocin-like GCF
Tab 2. Clinker links for BGC alignment of mycofactocin-like GCF
Tab 3. CBlaster hits for BGC alignment of mycofactocin-like GCF

Data S8. Results of IsaBGC-easy pipeline
Tab 1. Table of Conservation and Population-Genetic Statistics for Each Homolog Group Associated with the BiG-SCAPE GCF

References

1. Cantalapiedra,C.P., Hernández-Plaza,A., Letunic,I., Bork,P. and Huerta-Cepas,J. (2021) eggNOG-mapper v2: Functional Annotation, Orthology Assignments, and Domain Prediction at the Metagenomic Scale. *Mol. Biol. Evol.*, **38**, 5825–5829.

2. Huerta-Cepas,J., Szklarczyk,D., Heller,D., Hernández-Plaza,A., Forslund,S.K., Cook,H., Mende,D.R., Letunic,I., Rattei,T., Jensen,L.J., *et al.* (2019) eggNOG 5.0: a hierarchical, functionally and phylogenetically annotated orthology resource based on 5090 organisms and 2502 viruses. *Nucleic Acids Res.*, **47**, D309–D314.

3. Ondov,B.D., Treangen,T.J., Melsted,P., Mallonee,A.B., Bergman,N.H., Koren,S. and Phillippy,A.M. (2016) Mash: fast genome and metagenome distance estimation using MinHash. *Genome Biol.*, **17**, 132.

4. Ondov,B.D., Starrett,G.J., Sappington,A., Kostic,A., Koren,S., Buck,C.B. and Phillippy,A.M. (2019) Mash Screen: high-throughput sequence containment estimation for genome discovery. *Genome Biol.*, **20**, 232.

5. Jain,C., Rodriguez-R,L.M., Phillippy,A.M., Konstantinidis,K.T. and Aluru,S. (2018) High throughput ANI analysis of 90K prokaryotic genomes reveals clear species boundaries. *Nat. Commun.*, **9**, 5114.

6. Alanjary,M., Steinke,K. and Ziemert,N. (2019) AutoMLST: an automated web server for generating multi-locus species trees highlighting natural product potential. *Nucleic Acids Res.*, **47**, W276–W282.

7. Page,A.J., Cummins,C.A., Hunt,M., Wong,V.K., Reuter,S., Holden,M.T.G., Fookes,M., Falush,D., Keane,J.A. and Parkhill,J. (2015) Roary: rapid large-scale prokaryote pan genome analysis. *Bioinformatics*, **31**, 3691–3693.

8. Telatin,A., Fariselli,P. and Birolo,G. (2021) SeqFu: A Suite of Utilities for the Robust and Reproducible Manipulation of Sequence Files. *Bioengineering (Basel)*, **8**.

9. Kautsar,S.A., Blin,K., Shaw,S., Weber,T. and Medema,M.H. (2021) BiG-FAM: the biosynthetic gene cluster families database. *Nucleic Acids Res.*, **49**, D490–D497.

10. Kautsar,S.A., van der Hooft,J.J.J., de Ridder,D. and Medema,M.H. (2021) BiG-SLiCE: A highly scalable tool maps the diversity of 1.2 million biosynthetic gene clusters. *Gigascience*, **10**.

11. Parks,D.H., Imelfort,M., Skennerton,C.T., Hugenholtz,P. and Tyson,G.W. (2015) CheckM: assessing the quality of microbial genomes recovered from isolates, single cells, and metagenomes. *Genome Res.*, **25**, 1043–1055.

12. Chaumeil,P.-A., Mussig,A.J., Hugenholtz,P. and Parks,D.H. (2019) GTDB-Tk: a toolkit to classify genomes with the Genome Taxonomy Database. *Bioinformatics*, **36**, 1925–1927.

13. Parks,D.H., Chuvochina,M., Waite,D.W., Rinke,C., Skarshewski,A., Chaumeil,P.-A. and Hugenholtz,P. (2018) A standardized bacterial taxonomy based on genome phylogeny substantially revises the tree of life. *Nat. Biotechnol.*, **36**, 996–1004.

14. Seemann,T. (2014) Prokka: rapid prokaryotic genome annotation. *Bioinformatics*, **30**, 2068–2069.

15. Blin,K., Shaw,S., Kloosterman,A.M., Charlop-Powers,Z., van Wezel,G.P., Medema,M.H.

and Weber,T. (2021) antiSMASH 6.0: improving cluster detection and comparison capabilities. *Nucleic Acids Res.*, **49**, W29–W35.

16. Mungan,M.D., Alanjary,M., Blin,K., Weber,T., Medema,M.H. and Ziemert,N. (2020) ARTS 2.0: feature updates and expansion of the Antibiotic Resistant Target Seeker for comparative genome mining. *Nucleic Acids Res.*, **48**, W546–W552.

17. Alanjary,M., Kronmiller,B., Adamek,M., Blin,K., Weber,T., Huson,D., Philmus,B. and Ziemert,N. (2017) The Antibiotic Resistant Target Seeker (ARTS), an exploration engine for antibiotic cluster prioritization and novel drug target discovery. *Nucleic Acids Res.*, **45**, W42–W48.

18. Kim,G.B., Gao,Y., Palsson,B.O. and Lee,S.Y. (2021) DeepTFactor: A deep learning-based tool for the prediction of transcription factors. *Proc. Natl. Acad. Sci. U. S. A.*, **118**.

19. Navarro-Muñoz,J.C., Selem-Mojica,N., Mullowney,M.W., Kautsar,S.A., Tryon,J.H., Parkinson,E.I., De Los Santos,E.L.C., Yeong,M., Cruz-Morales,P., Abubucker,S., *et al.* (2020) A computational framework to explore large-scale biosynthetic diversity. *Nat. Chem. Biol.*, **16**, 60–68.

20. Carroll,L.M., Larralde,M., Fleck,J.S., Ponnudurai,R., Milanese,A., Cappio,E. and Zeller,G. (2021) Accurate de novo identification of biosynthetic gene clusters with GECCO. 10.1101/2021.05.03.442509.

21. Parks,D.H., Chuvochina,M., Rinke,C., Mussig,A.J., Chaumeil,P.-A. and Hugenholtz,P. (2022) GTDB: an ongoing census of bacterial and archaeal diversity through a phylogenetically consistent, rank normalized and complete genome-based taxonomy. *Nucleic Acids Res.*, **50**, D785–D794.

22. Blin,K., Shaw,S., Augustijn,H.E., Reitz,Z.L., Biermann,F., Alanjary,M., Fetter,A., Terlouw,B.R., Metcalf,W.W., Helfrich,E.J.N., *et al.* (2023) antiSMASH 7.0: new and improved predictions for detection, regulation, chemical structures and visualisation. *Nucleic Acids Res.*, **51**, W46–W50.

23. Terlouw,B.R., Blin,K., Navarro-Muñoz,J.C., Avalon,N.E., Chevrette,M.G., Egbert,S., Lee,S., Meijer,D., Recchia,M.J.J., Reitz,Z.L., *et al.* (2022) MIBiG 3.0: a community-driven effort to annotate experimentally validated biosynthetic gene clusters. *Nucleic Acids Res.*, **51**, gkac1049.

24. Buchfink,B., Reuter,K. and Drost,H.-G. (2021) Sensitive protein alignments at tree-of-life scale using DIAMOND. *Nat. Methods*, **18**, 366–368.

25. Gilchrist,C.L.M., Booth,T.J., van Wersch,B., van Grieken,L., Medema,M.H. and Chooi,Y.-H. (2021) cblaster: a remote search tool for rapid identification and visualization of homologous gene clusters. *Bioinformatics Advances*, **1**, vbab016.

26. Booth,T.J., Shaw,S. and Weber,T. (2023) Getphylo: Rapid and automatic generation of multi-locus phylogenetic trees. *bioRxiv*, 10.1101/2023.07.26.550493.

27. Gilchrist,C.L.M. and Chooi,Y.-H. (2021) clinker & clustermap.js: automatic generation of gene cluster comparison figures. *Bioinformatics*, **37**, 2473–2475.

28. Steinegger,M. and Söding,J. (2017) MMseqs2 enables sensitive protein sequence searching for the analysis of massive data sets. *Nat. Biotechnol.*, **35**, 1026–1028.

29. Raasveldt,M. and Mühleisen,H. (2019) DuckDB | Proceedings of the 2019 International Conference on Management of Data. In *Proceedings of the 2019 International Conference on Management of Data*, SIGMOD ’19. Association for Computing Machinery, Amsterdam Netherlands, pp. 1981–1984.

30. Salamzade,R., Cheong,J.Z.A., Sandstrom,S., Swaney,M.H., Stubbendieck,R.M., Starr,N.L., Currie,C.R., Singh,A.M. and Kalan,L.R. (2023) Evolutionary investigations of the biosynthetic diversity in the skin microbiome using lsaBGC. *Microb Genom*, **9**.

31. Gautreau,G., Bazin,A., Gachet,M., Planel,R., Burlot,L., Dubois,M., Perrin,A., Médigue,C., Calteau,A., Cruveiller,S., *et al.* (2020) PPanGGOLiN: Depicting microbial diversity via a partitioned pangenome graph. *PLoS Comput. Biol.*, **16**, e1007732.

32. Harke,A.S., Josephs-Spauling,J., Mohite,O.S., Chauhan,S.M., Ardalani,O., Palsson,B. and Phaneuf,P.V. (2023) Genomic insights into*Lactobacillaceae*: Analyzing the “Alleleome” of core pangenomes for enhanced understanding of strain diversity and revealing Phylogroup-specific unique variants. *bioRxiv*, 10.1101/2023.09.22.558971.

33. Hutchinson,M.C., Cagua,E.F., Balbuena,J.A., Stouffer,D.B. and Poisot,T. (2017) paco: implementing Procrustean Approach to Cophylogeny in R. *Methods Ecol. Evol.*, **8**, 932–940.
